# The “Big Six”: Hidden Emerging Foodborne Bacterial Pathogens

**DOI:** 10.3390/tropicalmed7110356

**Published:** 2022-11-07

**Authors:** Mona G. Alharbi, Rashad R. Al-Hindi, Ahmed Esmael, Ibrahim A. Alotibi, Sheren A. Azhari, Mazen S. Alseghayer, Addisu D. Teklemariam

**Affiliations:** 1 Department of Biological Sciences, Faculty of Science, King Abdulaziz University, Jeddah 21589, Saudi Arabia; 2 Nebraska Center for Virology, University of Nebraska-Lincoln, Lincoln, NE 68583, USA; 3 Botany and Microbiology Department, Faculty of Science, Benha University, Benha 13518, Egypt; 4 Health Information Technology Department, Applied College, King Abdulaziz University, Jeddah 21589, Saudi Arabia; 5 Monitoring and Risk Assessment Department, Saudi Food and Drug Authority, Riyadh 13513, Saudi Arabia

**Keywords:** *Escherichia coli*, non-O157, foodborne, Shiga toxin, reservoirs, outbreak

## Abstract

Non-O157 Shiga toxin-producing *Escherichia coli* (STEC) are emerging serogroups that often result in diseases ranging from diarrhea to severe hemorrhagic colitis in humans. The most common non-O157 STEC are O26, O45, O103, O111, O121, and O145. These serogroups are known by the name “big six” because they cause severe illness and death in humans and the United States Department of Agriculture declared these serogroups as food contaminants. The lack of fast and efficient diagnostic methods exacerbates the public impact of the disease caused by these serogroups. Numerous outbreaks have been reported globally and most of these outbreaks were caused by ingestion of contaminated food or water as well as direct contact with reservoirs. Livestock harbor a variety of non-O157 STEC serovars that can contaminate meat and dairy products, or water sources when used for irrigation. Hence, effective control and prevention approaches are required to safeguard the public from infections. This review addresses the disease characteristics, reservoirs, the source of infections, the transmission of the disease, and major outbreaks associated with the six serogroups (“big six”) of non-O157 STEC encountered all over the globe.

## 1. Introduction

*Escherichia coli* (*E. coli*) is one of the major intestinal commensals of the human gastrointestinal tract (GIT) [1]. The pathogenic strains of *E. coli* strains can cause several diseases such as meningitis, bacteremia, wound infections, urinary tract infections, and diarrhea in humans [2]. The *E. coli* strains that cause diarrhea are referred to as diarrheagenic *E. coli* (DEC) [3,4,5], and six types of DEC have been recognized based on their virulence factor and serotypes including Shiga-toxin producing *E. coli* (STEC) or enterohemorrhagic *E. coli* (EHEC), enterotoxigenic *E. coli* (ETEC), enteropathogenic *E. coli* (EPEC), enteroinvasive *E. coli* (EIEC), enteroaggregative *E. coli* (EAEC) and diffuse adherent *E. coli* (DAEC) [6].

STEC produces Shiga toxins (*stx1* or *stx2*) that can cause local colon damage, leading to hemorrhagic colitis (HC), and complications such as hemolytic-uremic syndrome (HUS) in humans [7]. STEC bacteria that cause human disease typically contain a locus of enterocyte effacement (LEE), which facilitates the formation of attaching-and-effacing (AE) lesions. LEE encodes intimin, an outer membrane protein, which facilitates the attachment between enterocytes and bacteria [7,8]. Some serogroups of *E. coli* possess more than one strain as in the case of O26 serogroups which comprised both STEC and EPEC strains. In this regard, there are two types of EPEC strains: typical EPEC (tEPEC) and atypical EPEC (aEPEC) [9]. These two groups share the pathogenicity island LEE and can cause AE lesions. Nevertheless, only tEPEC has the EPEC adherence factor plasmid (EAF plasmid), which encodes bundle-forming pilus (BFP) (encoded by *bfpA*) [9]. aEPEC, however, often possesses enteroaggregative *E*. *coli* heat-stable enterotoxin 1 (EAST1) (encoded by *astA*) [7,9].

Among the several strains of *E. coli*, O157:H7 is the most frequently isolated serovar because of the seriousness of the prognosis of the disease it causes [10]. It has been reported that these strains of *E. coli* (O157:H7) can produce Shiga toxins (*stx*) that lead to serious illness in humans. Humans have been infected with over 400 different STEC serotypes of *E. coli*. Over 100 of these serogroups have been linked to human GIT infections ranging from mild diarrhea to HC and severe HUS [11]. Aside from *E. coli* O157:H7, other *E. coli* serotypes produce these toxins and cause foodborne illness.

The US Food and Drug Administration (FDA) has identified six serogroups, known as the “big six”: *E. coli* O26, O45, O103, O111, O121, and O145. These six serogroups are the most frequently identified serotypes associated with foodborne illness in different countries. In the US, these serogroups cause foodborne sickness for nearly 169,600 individuals each year [12].

In Africa, several non-O157 serotypes including the most prevalent serotypes such as O26, O111, O103, and O145 have been isolated from different parts of the continent (Central, East, North, South, and West). In Egypt, for instance, STEC O158, O125, O114 and O26 have been isolated from chickens, cattle, humans, sheep, and water [13,14]. In Tanzania, STEC O113 has been isolated from feces of cattle [15]. In South Africa, detection of STEC isolates from diarrheic individuals displayed the presence of O157 and non-O157 STEC including O111, O84, O26, O21, O5 and O4 (72). In the same country, STEC isolates that caused human disease between 2006 and 2013 were mostly non-O157 strains (STEC O26:H11, O157:H7, O111:H8, and O117:H7) which had virulence genes (*stx1*, *eae*A, *ehxA* and *espP*) commonly linked to STEC strains that have been responsible for mild to severe disease in humans all over the globe [16].

In Australia, according to the report by Vally et al., STEC infections occurred steadily for the 11-year period from 2000 to 2010 and the estimated incidence rates were found to be lower or comparable to the levels recorded in European countries. During this period O157 strains, and among the non-O157 strains O26 and O111, caused STEC infections [17]. In 2017, in Argentina, Brusa et al. studied the prevalence of non-O157 STEC isolated from beef samples collected from eight cattle slaughterhouses. The results revealed that the prevalence of non-O157 STEC was low, and the most prevalent serotypes were O8:H19, O130:H11, O174:H21, O178:H19, and O185:H7. All O103:H21 strains and one O178:H19 strain were positive for *eae* and *aggR/aaiC*, respectively [18].

According to the European Food Safety and Authority (EFSA), it is notable that STEC confirmed cases in the EU increased between 2015 and 2019, but the overall trend for STEC from 2016 to 2020 has not shown any significant changes. In 2020, forty-eight different non-O157 serogroups were reported, with the most prevalent being O146 (13 isolates), followed by O38 (seven) and O6 (four strains) [19]. An epidemiological study conducted from 2013 to 2017 on STEC in the Southeast of England showed that one among the big six serogroups and three other non-O157 STEC serogroups (O91, O157, and O146) caused non-travel-linked STEC infection. Of all, 76% of non-O157 isolates harbored the *eae* virulence gene [20].

It has been noticed that the big six serogroups share many virulences and epidemiological features with *E. coli* O157:H7. Several reports suggest that undercooked and contaminated food and water as well as person-to-person transmissions within families serve as vehicles in non-O157 outbreaks. In sporadic outbreaks, direct contact of individuals with cattle has been identified as a risk factor [21]. In most cases, beef, milk, cheese, and juice are the food commodities that are linked to these outbreaks [22,23]. All non-O157 STEC strains except O45 have been associated with HUS [24]. The infectious dose is a few hundred cells or lower than the dose caused by *E. coli* O157:H7 [24]. Several virulence factors have been reported to be involved in both non-O157 STEC and *E*. *coli* O157:H7 infections and among these the most important factors are intimin and Shiga toxins 1 and/or 2 (*stx1*, *stx2*) [25].

Non-O157 STEC serogroups lack specific biochemical markers that can be used for screening and identification of the agent from food, stool, or other representative samples [26]. Hence, there is a lack of rapid and simple diagnostic methods for accurate and efficient detection of non-O157 STEC. As a result, the magnitude of the disease caused by these serogroups is likely underestimated (hidden) all over the globe [26].

This review provides an overview of the six non-O157 *stx* producing *E*. *coli*, the disease characteristics, reservoirs, the source of infections, the transmission of the disease, and major outbreaks associated with the six serogroups encountered all over the globe.

## 2. Characteristics of Diseases Caused by Non-O157 STEC Infections

In the US, non-O157 associated infections caused by six serogroups (“big six”) account for 80 percent or more of non-O157 STEC infections. These serogroups are O126, O111, O103, O121, O45, and O145 [27]. The incubation period of diseases that are caused by O157 and non- O157 serotypes is usually from 3 to 4 days; however, it can be as short as 1–2 days or as long as 5–8 days. The disease process of STEC is initiated by overriding the intestine’s natural defense strategies. The ability to tolerate acidic environments is one of the characteristics of STEC that allows the agents to remain active in the acidic environment. Those serovars which harbor the *eae* (intimin or attaching and effacing (EF)) gene can express proteins that facilitate cellular attachment. At the time of attachment, strains that are positive for *eae* produce an AE lesion on the intestinal epithelial cells. The AE lesion leads to typical morphological alterations in the epithelial cells of the infected individuals, such as pedestal formation, loss of microvilli, and aggregation of cytoskeletal proteins, which eventually result in firm attachment of the causative agents onto the surface of the host cell. Once adhered, *stx* is internalized into the host cell via cellular trafficking and causes systemic disease [28,29].

Primary clinical signs include a short-term fever, abdominal pain, and non-bloody diarrhea. The diarrhea stage of sickness is accompanied by vomiting but is displayed in only about half of the infected individuals. Bloody diarrhea (BD) may be seen in the first couple of days following infection with pronounced abdominal pain. In some cases, this situation may persist for up to 10 days. The majority of STEC infections will disappear without any complications; however, 10% of infected individuals, mainly the elderly and young individuals (under 10 years old) may experience HUS [30,31,32]. HC is exemplified by serious abdominal pain accompanied by watery diarrhea, followed by BD with minor fever or without fever. BD is more frequently encountered in patients who are infected with the O157:H7 serotype than in those who are infected with non-O157 STEC. HUS was detected in 1955 and associated with *Shigella dysenteriae* toxins. HUS in humans is accompanied by thrombocytopenia, renal failure (usually acute), and microangiopathic hemolytic anemia, which leads to renal tissue and intestinal damage [32]. It has been reported that approximately 80% of HUS cases are caused by O157 STEC infections, while <10% of HUS cases are associated with non-O157 STEC [33,34]. The pathogenic serogroups especially the “big six” serovars of non-O157 STEC can cause BD; however, only the serotypes which harbor *stx2* usually cause HUS. Some *stx* non-O157 serogroups, such as serotypes O111 and O26, have been linked to HUS and HC [35]. Clinical symptoms of illness that have been caused by non-O157 STEC are similar to those caused by O157:H7 serotypes [35]. In some studies, the magnitude of illness associated with non-O157 infection has been just as severe as diseases caused by O157:H7 serotypes [29,36].

## 3. Major Virulence Factors

### 3.1. Shiga toxin (Stx)

Shiga toxin (*stx*) was first detected in 1977 [37]. *E. coli* chromosomes do not encode *Stx* but their genes (*stx* genes), are encoded by genomes of phages which present as prophages in their hosts. The *stx* phages belong to the lambdoid phage group [38]. Phage DNA can passively replicate together with the host genome once it has been integrated into the bacterial chromosome [38].

PCR may reveal the existence of STEC *E. coli* in samples via the detection of *Stx* gene (s), but often, the successive isolation of the organism from culture may lead to negative results. This inconsistency could be allied to low cell concentration, existence of dead or injured cells, competitive microflora, and cells in a viable but non-culturable state, and the presence of free DNA, fragments of *stx* genes, and intact phages which can harbor the *stx* gene leading to culture-negative/PCR-positive results [39].

STEC serotypes are typified by their capability to express either one or two cytotoxins, referred to as *stx1* (VT1) and *stx2* (VT2) [40]. The *stx* are categorized into two classes, *stx1* (analogous to the *stx* produced by *S. dysenteriae* Type 1) and *Stx2* [41]. *stx1* and *stx2* have numerous clinically relevant subtypes [41]. Various *stx2* subtypes are designated by suffix letters and among these subtypes, *stx2a* (with or without *stx2c*) is responsible for HUS [42]. Unlike elastase-activated *stx2d*, other serogroups which do not depend on this protein result in milder disease [43]. Two subtypes, *stx2*e and *stx2*f, have been detected from serovars that infect animals and are seldom detected from human infecting *E. coli*. In vitro Vero monkey kidney cells-based studies [44] indicated that *stx2*a, *Stx2d*, and *stx2d* (elastase activable) are more potent toxins than *Stx2c* [43]. A pair of toxins, namely *stx1* and *stx2b*, are the least potent toxins among all subtypes. According to epidemiological studies, STEC serogroups that harbor one *stx2* gene are more likely to cause HUS than those strains which possess two of the toxigenic genes (*stx1*a and *stx2*a) [44]. Both *stx1*a and *stx2a* toxins can pass the barrier of the intestine at an equal speed and accumulate in the kidneys. The combination of *eae* gene and *stx2a* has been linked to the development of BD and HUS in infected individuals [45].

### 3.2. Enterocyte Effacement (LEE) Pathogenicity Island

The LEE pathogenicity island of the non-O157 genome encodes a type III secretion needle which assists in the AE lesions development on the mucosa of the colon [46]. Intimin is one of the crucial proteins encoded by the *eae* gene, which contributes to both the colonization of bacteria on the epithelium of the intestinal layer and AE lesions. Some of the STEC serovars may comprise both intimin and *stx2* and these most commonly lead to severe disease in humans. According to some outbreak investigations, LEE most likely confers greater virulence, since LEE-positive STEC serogroups (O157:H7, O111:NM, O26:H11, O121:H19, O103:H2, and O145:NM) are more strongly related to HUS and other severe cases than outbreaks caused by LEE negative serogroups [47].

### 3.3. Plasmid-Encoded Enterohemolysin

Hemolysin is one of the virulence factors of STEC serotypes which play a substantial role in the progression of STEC infections. Currently, four types of hemolysin (*ehxA*, *hlyA*, *e-hlyA*, and *sheA*) have been detected in *E. coli*. Among these hemolysins, the plasmid-encoded enterohemolysin (*ehxA*) is prevalent in STEC serotypes and is often correlated with HUS and diarrheal cases [48]. The involvement of enterohemolysin in STEC infections is directly linked to *stx* production; thus, it has been considered an epidemiological marker for the simple and fast detection of STEC serotypes [49]. Six genetically unique subtypes of *ehxA* (designated from A to F) were detected in *E. coli* using PCR or other advanced molecular detection tools [48]. The whole *ehxA* gene measures 3.0 kilobases and is located in the *ehx* cluster [50] wherein *ehxC* is responsible for the activation of hemolysin, and *ehxB* and *ehxD* are related to the secretion of hemolysin [50]. It is believed that these genes play an imperative role in the STEC disease process; however, their functions are not fully elucidated [51].

## 4. Reservoir, Source, and Transmission of Non-O157 *E. coli*

Animals, mainly ruminants, are the potential reservoirs for both non-O157 and O157:H7 STEC. According to scientific reports, non-ruminants such as pet psittacine birds (budgerigars, cockatiels, etc.), have carried non-O157 STEC in their GIT system [52]. In Australia, for instance, there were two non-O157 STEC outbreaks reported associated with non-ruminants: an outbreak in 2002 involving pigs and another involving alpacas with STEC serotype O26 at a petting zoo [53].

There has been evidence that cattle shed STEC more frequently in warmer months than in colder months, and this correlates with an increase in human illness during the summer [31,32]. Additionally, age has been found to affect the fecal shedding of STEC in cattle. Calves before weaning shed STEC at the lowest rates, while postweaning cattle shed at the highest rates, and adult cattle shed STEC at intermediate rates [31]. Research findings indicate that many non-O157 bovine isolates are unlikely to harbor virulence traits other than *Stx*, such as hlyA and *eae*, suggesting that they may not be as virulent as the non-bovine isolates [54,55]. Small ruminants such as sheep are known to be a potential STEC reservoir, responsible for the dissemination of STEC in the environment, herd, and slaughtering houses as well [56]. In a study conducted by Shahzad and colleagues indicated that both buffalo and sheep samples harbored rfbE O157 genes, while unlike the sheep samples, rfbE O157 isolate of buffalo samples encoded 4 STEC virulent genes (*Sxt1*, *stx2*, *ehlyA* and *eae*), suggesting that buffalo and adult healthy sheep are probably indispensable carriers of STEC O157 and reflecting a potential source of STEC contamination to humans and environment [57].

McCarthy et al. studied the prevalence of STEC *E. coli* in slaughter-age Irish sheep and found a high STEC prevalence circulating within the younger animals and sheep population and that STEC carriage is frequently encountered during the summertime. Among the thirty-five serotypes reported in this study, fifteen were new serotypes which had not been reported for sheep. O91:H14 was the most prevalent serotype encountered. Based on the results the authors suggest that sheep shed the organism through feces and are overwhelmingly recognized as potential contributors to environmental STEC contamination [58].

Generally, ruminants may shed huge amounts of STEC for long time and this scenario is named as “super-shedding,”. It has been realized that super-shedding constitutes the supreme source of STEC contamination in the agri-food supply chain [59]. Both non-O157 STEC and *E. coli* O157 serogroups have been isolated from super-shedding ruminants [60,61]. According to McCarthy et al., postharvest interventions and carcass dressing significantly reduced the occurrence of these pathogen along the food supply chain as it is reported in case of sheep [62].

**Figure 1:**
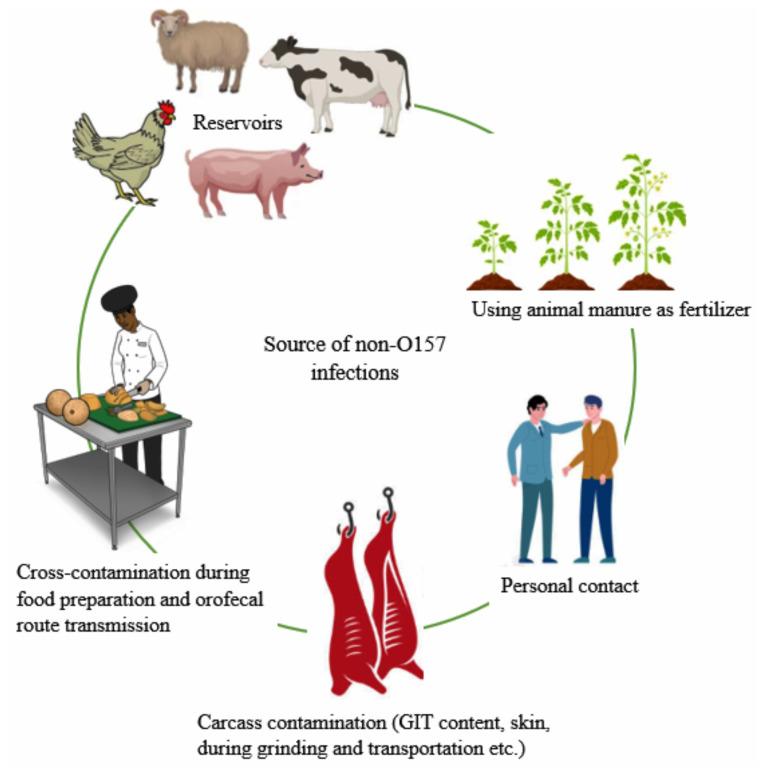
The major source of infections associated with non-O157 *E. coli* infection.

There are similarities between outbreak sources (Figure 1) and risk factors for non-O157 STEC and *E. coli* O157:H7. Human infection mainly occurs through the consumption of food products that contain cattle excrement or water contaminated with it [63]. It has been demonstrated that poorly prepared or handled ground beef can cause STEC infection, and as a result, the US Department of Agriculture and Food Safety Inspection Service (USDA-FSIS) declared O157:H7, and STEC O145, O121, O111, O103, O45, and O26 (collectively known as the top 7 STEC) as food adulterants with zero tolerance for raw ground beef and beef products [64]. Meat that has been contaminated with STEC during slaughter is the critical route through which the “big six” pathogens enter the meat processing plants. Ground beef can be contaminated during grinding activities, making it one of the high-risk meat cuts among retail meat cuts [65]. According to research findings, meat and water-associated outbreaks caused by non-O157 STEC serogroups are less common than STEC O157 [66]. Apart from meat, contaminated dairy products, such as unpasteurized cheese, have caused several outbreaks, as in the case of STEC O26:H11 in Italy and Romania [67]. The consumption of raw or insufficiently pasteurized milk [24,63], contact with animals and their manure [68], consumption of fresh products contaminated with animal manure [69], and exposure to a contaminated swimming pool [17,68] have also been identified as risk factors. Humans can also acquire the infection from fecally contaminated rivers, lakes, and municipal wastewater [17,68].

Human-to-human transmission of STEC serogroups is reported to be common in childcare centers. An outbreak caused by STEC serogroup O45 (*stx1*) has been reported in a prison in 2006 [70], whereby an infected food handler was found to be the source of infection.

## 5. Six Serogroups of Non-O157 Shiga toxin-producing *E. coli*

### 5.1. E. coli Serogroup O26

Serogroup O26 *E. coli* possesses both EPEC and STEC strains. STEC O26 is the most common non-O157 serogroup related to HUS and HC in humans [71], while EPEC O26 is responsible for less-severe enteritis [72]. The human isolates of *E. coli* O26 mostly express the flagellar (H) antigen (e.g., H11) or are nonmotile (NM/H-) if the antigen is not encoded by their genome. Nevertheless, based on the molecular assessment, it has been proven that the H-serogroup is also classified under the H11 clonal complex [73]. EPEC O26:H11 does not comprise the EAF plasmid and is hence categorized as aEPEC [74]. However, classifying O26 serogroup into pathogroups, such as STEC and aEPEC, may be misleading, since aEPEC could be STEC which lacks *stx* and the reverse [75].

O26 serogroup has been isolated from both healthy as well as diarrheic animals [76,77,78]. Even though myriad studies on O26 have been performed, most of these investigations have been inadequate, with limited sample sizes, or have centered on STEC O26 rather than non-STEC O26 serotypes. Besides, most studies have been conducted on cattle [79,80], and research on O26 in sheep is scarce [77,81]. Among the STEC serogroups, O26 is the second most reported serogroup in several countries, including Ireland, Italy, France, and Denmark, and clinical O26 cases presently exceed cases caused by O157 [82]. Non-O157 infections were found to be clinically relevant in pediatric patients, in whom the disease severity is comparable to that of O157 [83,84]. Current outbreaks of *eae* and *stx2* positive O26:H11 serogroup have caused severe HUS infections in young individuals, particularly in some countries, including France, Italy, and Romania [85,86,87,88]. To date, in European countries, the O26:H11 serogroup is accountable for more HUS infections than O157 STEC serogroup and is of emerging importance [82].

### 5.2. E. coli Serogroup O45

STEC O45 serogroup is one of the top six non-O157 STEC that have been recognized as a cause of sporadic BD in humans [89]. During the first outbreak of STEC O45 in 2005, 52 inmates in New York City became ill with diarrhea or BD, probably because they had been exposed to an ill food worker [90]. In addition to these outbreaks, two other outbreaks caused by O45:H2 serogroup have occurred in which 18 illnesses were reported, and contaminated smoked goat and game meat was implicated as the source of contamination in both outbreaks [91]. In a different study, two O145:H28 strains (RM13716 and RM13714) caused ice cream and lettuce-associated outbreaks in Belgium and US, respectively. These strains shared a common ancestor with 5 different STEC O157:H7 strains, including the Japanese *E. coli* Sakai strain [92,93].

### 5.3. E. coli Serogroup O103

Among the other STEC serogroups of *E. coli*, O103 has caused a few outbreaks worldwide. STEC O103:H2 is one of the most prevalent STEC serotypes isolated from humans in Europe [94]. *E. coli* O103:H11 has been reported in Japan and Canada as a sporadic cause of human infections in addition to *E. coli* O103:H2 [95].

An *E. coli* outbreak caused by O103 occurred in Norway in the spring of 2006. Among the 17 cases identified, 10 children experienced HUS and one died. The outbreak was traced to a cured sheep sausage product from one brand [96]. S*tx*-producing *E. coli* O103 H2 was first isolated in Brazil from sheep in 2004 and re-emerged in 2005 [97]. During the period 1997–2000, STEC *E. coli* O103 H2/H (-) ranked third among the most repeatedly isolated EHEC types in Germany [98]. The pathogen has also been reported in sporadic outbreaks at nursery facilities in Japan between 2010 and 2013 [99]. Recent outbreaks of *E. coli* O103 have been reported in Germany following school-led trips to Austria [100]. In this case, raw cow’s milk was implicated as the source of the infection, and international collaboration played an essential role in preventing outbreaks and responding appropriately.

### 5.4. E. coli Serogroup O111

Serogroup O111 causes enteropathogenic and enterohemorrhagic sickness in humans [32]. EPEC O111 serogroup is the leading cause of diarrhea in infants, predominantly in developing nations. Among the non-O157 *E. coli* serovars, *stx*-producing EHEC O111 is one of the frequent causes of BD and HUS across the world [101,102]. Many outbreaks have been correlated to this pathogen [89,101].

Research on the O111 serogroup that has originated from different sources indicated that there is a substantial phenotypic and genetic diversity among this *E. coli* O serogroup. Even though motile O111 serotypes isolated from children’s diarrhea usually expressed flagella antigens (H2, H12, or H21), in several cases NM isolates have been encountered. Serogroup O111:H and O111:H2 characteristically carry the EAF plasmid that facilitates localized attachment (LA) of bacteria to cultured cells which is a feature of the classic EPEC serogroups.

The O111 clone was the first STEC serogroup of *E. coli* that cause gastroenteritis outbreaks in humans [103]. The flagellar type O111:H12, O111:H8, O111:H2, and the non-flagellated O111: NM has been recognized as pathogenic clones. The O111 antigen has been classically linked to the enteropathogenic serogroup and now has also been recognized as an O antigen of EHEC and EAEC *E. coli* [32]. Due to the lack of facilities for proper diagnosis of O111 serotype of *E. coli*, the burden of the public health illness caused by these clones is underestimated. However, several O111 serotypes have been reported as they caused serious enteric illness in humans, including 28% of 50 outbreaks of child diarrhea in the US from 1934 to 1987 [104], 33% of infantile diarrhea cases in Brazil [105], a huge outbreak affecting >700 infected individuals in Finland [106], and currently, documented HUS outbreaks in Italy [107] and Australia [108].

### 5.5. E. coli Serogroup O121

S*tx*-producing O121 *E. coli* serogroups have been mainly isolated from individuals who developed HUS or HC, and consequently they are categorized as EHEC [109,110,111]. Moreover, these serogroups having virulence factors like those of enteroinvasive *Shigella* and *E. coli*, have resulted in shigellosis-like sicknesses [112]. In 1999, an HUS outbreak caused by O121:H19 serogroup was reported and the source of the infection was related to the lake in Connecticut [109]. Tarr et al. identified 24 isolates of O121:H19 serogroup and H- (NM) variants using multilocus sequencing and enzyme electrophoresis and the results indicated that the isolates were determined as a single bacterial clone. These isolates comprised a virulence gene that is specific for EHEC clones; nevertheless, the sequence analysis indicated that the O121:H19 clone was not classified under either EPEC *E. coli* or classical EHEC groups. Tarr et al. indicated that O121:H19 serogroup gained virulence genes and denotes a typical EHEC clone [113].

### 5.6. E. coli Serogroup O145

O145 is a crucial cause of HUS and HC worldwide. Ruminants are the main reservoir of this serogroup, which carry the agent in their hind GIT system and shed it in the manure. Fecal matter is the major source of carcass and hide contamination which leads to O145-associated foodborne infections in humans. Several outbreaks have been reported associated with O145 infection in the US and other countries, including Argentina [114], Germany [115], and Belgium [116]. In 1999, two O145-associated illnesses were reported in a daycare center in Minnesota, US [22]. This serogroup has also been reported as the cause of a waterborne infection in humans in 2005, in Oregon, US [117], and in 2010, the consumption of contaminated romaine lettuce resulted in a multistate outbreak, causing 45% hospitalization, associated with HUS in 10% of cases [118].

## 6. Outbreaks Caused by Non-O157:H7 Infections

An outbreak is defined as epidemiologically associated illnesses (clustered in space or time) with two or more individuals who are positive for culture-confirmed non-O157 STEC infection. The term single-etiology outbreak is used to describe outbreaks where only a single serogroup has been isolated. A multiple-etiology outbreak is defined as the isolation of only one non-O157 STEC serogroup from at least two ill individuals, and evidence of another enteric pathogen from at least two sick individuals [119].

Most deadly STEC outbreaks reported worldwide have been caused by STEC O157:H7. Nevertheless, non-O157 serotypes have emerged in recent years as significant enteric pathogens across countries such as Argentina, Japan, Chile, Germany, the United States, Australia, and Ireland [120]. Several types of food commodities including water are the main source of infection for most large outbreaks of non-O157 STEC. It has been reported that sources associated with O157 outbreaks have also been implicated in non-O157:H7 outbreaks. Since 2010, most of the outbreaks have been attributed to both lettuce and sprouts [12]. Nevertheless, milk, ground beef, and water are also ideal vehicles for non-O157:H7 infection [121,122]. Livestock farms run off has been recognized as the major source of contamination in agrarian land; however, microbial contamination can also occur at the time of harvesting and processing. Due to the difficulty in accurate diagnosis of non-O157:H7 STEC serotypes, many other outbreaks may not have been reported. Table 1 summarizes published data on the major non-O157 STEC outbreaks by country, causative agent, source of infection, and reported cases.

## 7. Diagnostic Approaches

### 7.1. Cultural Methods

Unlike non-O157 strains, serogroup O157 are not sorbitol fermenters and are readily detected by culture, producing colorless colonies on sorbitol MacConkey (SMAC) agar [132]. In this agar, lactose which is the principal component of typical MacConkey agar is replaced by sorbitol, whereby the non-sorbitol fermenter appeared colorless on the medium [132]. Previous studies found that SMAC agar showed a satisfactory level of specificity, sensitivity, and negative predictive value for the detection of *E. coli* O157:H7 from different samples; nevertheless, its positive predictive value was 28%. Moreover, this medium could not detect the isolates which harbor the *stx* genes for both sorbitol-fermenting O157 and non-O157 STEC isolates [133,134]. CHROMagar^TM^ O157 is one of the first chromogenic media that have been developed for the detection of STEC. In this culture medium, O157 STEC produces mauve colonies while other *E. coli* appear blue. CHROMagar™ O157, like SMAC agar, is not efficient to detect most non-O157 STEC [135]. Rainbow^®^ Agar O157 is the other chromogenic medium that has been prepared to detect non-O157 serotypes (O26:H11, O48:H21, O111:H8, O111:H-), and O157:H7 serogroups based on their reduction or lack of β-glucuronidase activity in comparison to non-toxigenic serotypes. Rainbow^®^ Agar O157 has been assessed in numerous studies for the detection of STEC in water and food [136,137]. Its role in detecting STEC from stool was initially studied at the Alberta ProvLab. Zelyas and his colleagues evaluated four chromogenic differential media for their efficacy to detect non-O157 STEC at the Alberta ProvLab [138]. A total of 161 non-O157 STEC isolates were tested for their growth on Rainbow^®^ Agar O157, CHROMagar™ STEC, Colorex^®^ O157, and CHROMagar™ O157, to see which media support the growth of the tested isolates. Unlike Colorex^®^ O157 and CHROMagar™ O157 which were not able to identify most of the non-O157 isolates, Rainbow^®^ Agar O157 and CHROMagar™ STEC supported their growth with detection rates of 70% and 90%, respectively.

### 7.2. Immunological Methods

Due to the lack of convenient culture media for detecting all STEC serotypes, identifying the *Stx*s may provide an alternative method of diagnosing STEC-related infections [139]. In this regard, researchers tested different types of immunological methods such as microwell, optical immunoassay, and immunochromatographic which had different levels of the detection limit. Premier^®^ EHEC microwell immunoassay is one of the enzyme immunoassays (EIAs) that has been developed for the detection of toxigenic serotypes. Several studies conducted on stool samples with the involvement of enrichment culture indicated that Premier^®^ EHEC displayed high specificity and sensitivity in detecting toxigenic serotypes. One study found that Premier^®^ EHEC was highly sensitive (83.9%) and specific (99.8%) for detecting *stx* directly from samples without enrichment [140].

In addition to microwell EIAs, other immunoassays have been introduced to detect STEC. Among these assays, the BioStar^®^ SHIGATOX optical immunoassay detects the toxins by its immunological communication with anti-*stx* antibodies coated on a silicon wafer surface. The interaction causes the thin film’s optical thickness to increase, resulting in a change in color on the wafer. Likewise, the Duopath Verotoxin-test™ is an immunochromatographic assay developed by mounting anti-*stx* antibodies on a membrane for specific binding and detection of *stx*. According to former laboratory investigations, the BioStar^®^ SHIGATOX assay displayed greater cultivation potential than the Duopath Verotoxin-test^TM^. Nevertheless, it is advantageous to use the Duopath Verotoxin-test^TM^ since it can distinguish between *stx1* and *stx2*-producing serovars.

Furthermore, the Alberta ProvLab assessed two immunochromatographic assays: Shiga Toxin Quik Chek™ and ImmunoCard STAT!^®^. Despite its high specificity (>99%), ImmunoCard STAT!^®^ had a low sensitivity of 35.5% even in the presence of enrichment broths. Shiga Toxin Quik Chek™ displayed sensitivities of 70% and 85% without and with enrichment, respectively [141,142].

### 7.3. Molecular Methods

Several PCR assays have been established for the detection of non-O157 *E. coli*. Most of these assays are used in real-time setups. Real-time PCR (RT-PCR) has several advantages over conventional PCR including high specificity and sensitivity and it is able to detect and distinguish between *stx1* and *stx2*, other virulence genes (*eae*, *ehx4*), and genes of other GIT pathogens. The first reported RT-PCR used directly on clinical stool for detection of *Stx* had a specificity of 92% and a sensitivity of 100% [143]. The GeneDiscCycler (Pall/GeneSystems, Bruz, France) is one of the RT-PCR platforms which can detect and identify various STEC serogroups. This PCR platform provides simultaneous identification of *eae*, *Stx1*, *stx2*, and O-group antigens O157, O26, O111, O103, and O145 [144]. The GeneDiscCycler protocol includes sample enrichment, DNA extraction, and amplification of *eae* and *stx* genes, followed by serotyping. Similarly, Macori and colleagues used a real-time PCR assay for the detection and enumeration of *E. coli* O157 and O26 serogroups in sheep recto-anal swabs [145].

### 7.4. Flow Cytometry

Most of the conventional serotyping methods developed for the detection of “O” groups are resource and labor-intensive and can take at least 5 days to get the results. However, researchers have developed fast and efficient approaches for the screening of “O” groups of non-O157 STEC serotypes [146,147]. Flow cytometry is one of these emerging approaches that may be manipulated for fast detection of this antigen for clinical cases, food safety, environmental monitoring, and medical diagnosis [147,148]. In this line, Hegde and colleagues developed highly specific, sensitive, and rapid flow-cytometric assays for the detection of O groups of the big six non-O157 STEC in ground beef. The detection limit of the developed assay was 1 to 10 CFU and its sensitivity was 2 × 10^3^ in 10^5^ CFU/mL bacterial mixture [149]. In this assay, there was no report of cross-reactivity between the O antigen of the six STEC serogroups and other serogroups or species of bacteria tested. Hence, this method may be employed for quick identification of the big six serogroups coupled with PCR for intimin and *stx* genes detection [149].

### 7.5. Metagenomics

Whole genome sequencing (WGS) allows efficient detection, identification, and confirmation of STEC isolates at single nucleotide level, making it the decisive tool for regular surveillance and outbreak investigation. Nevertheless, this method suffers from lack of standardization, and the disparity concerning bioinformatics parameters and roadmaps, which hampers interoperability among national research institutions [150]. In this regard, Lang et al. introduced a validation approach linked to a bioinformatics platform for Illumina that works on WGS-based characterization of *Stx*-producing STEC isolates including virulence gene detection, prediction of antimicrobial resistance and serotype, sequence typing and detection of plasmid replicon which result in high performance with reproducibility, repeatability, precision, accuracy, specificity and sensitivity over 95% for the majority of all assays [150]. The employment of WGS boosts the detection and complete characterization of non-O157 STEC strains. WGS resolves the unreliable PCR detection of serogroup-specific and *stx1*, *stx2* genes including from nontypeable strains and less frequently isolated STEC serovars [151].

A comparative study on pulse field gel electrophoresis (PFGE) and WGS for characterization of STEC O26 strains revealed that out of the seven *E. coli* strains, six isolates serotyped phenotypically and by WGS as *E. coli* O26:H11, while one bovine isolate serotyped only by WGS as *E. coli* O182:H25. *stx1* was identified in bovine and human isolates using PCR and WGS. In contrast, four STECO26 isolates were indistinguishable by PFGE [152]. Another comparative study indicated that WGS-based detection of STEC showed a very high degree of overlap with conventional or classical methods. Specifically, of the typeable strains, the rate of concordance was 99% and 97% for H and O antigens, respectively and >99% for *stx1*, *stx2*, or eaeA for all strains. Of the nontypeable strains, 99% and 100% O and H antigens were identified by WGS, respectively [151].These results indicated that WGS offered more reliable information than traditional conventional tools especially from a public health perspective.

In a different comparative study conducted by Castro et al., out of the thirty-nine *E. coli* strains analyzed by PCR and WGS, eleven strains were confirmed by WGS as STEC containing full *stxA* and *stxB* subunits. Nonetheless, shortened *stx* fragments were detected in twenty-two isolates, whereby some comprised multiple *stx* fragments. Isolates with complete *stx* by WGS had consistent *stx1* and *stx2* detection by PCR except one *stx2*. For all STEC and eighteen non-STEC strains, serogroups determined by PCR were consistent with those determined by WGS. However, two WGS isolates were *Citrobacter* spp. and three other serotypes were indecisive. In this study, of all the thirty-nine strains, *stx* prophage was encountered in fourteen isolates but they were fragmentary or incomplete, probably because of partial excision of phage following sub-cultivation or other unknown reasons [153]. In a different study, Yang et al. used WGS and identified *stx2* from five STEC strains recovered from raw beef and mutton in China. The results showed that all the *stx2*l-STEC strains categorized to the O8 serogroup. *stx2*l-converting prophages from several sources shared a significant sequence similarity and structure [154].

## 8. Prevention and Control of Non-O157:H7 *E. coli* Infections

Several efforts have been made to establish strong animal herd management and food safety practices to prevent the occurrence of O157:H7 and non-O157 infections. To this end, the application of hazard analysis critical control points and post-slaughter decontamination practices in slaughter plants has been reported to considerably reduce the contamination of carcasses and organs with the above-mentioned pathogens [155]. Pre-slaughter interventions in cattle at the lairage can prevent the introduction of O157:H7 and non-O157 into the food processing chain. Intervention strategies aimed at reducing the pathogenic microbial load of live animals and/or carcasses before and after slaughter have been developed [156]. As part of an integrated multiple-hurdle approach to minimizing pathogen contact and subsequent human illnesses, live-animal interventions have been implemented in a coherent, complementary manner to post-therapeutic approaches [157].

The use of vaccines at the farm level can counteract or abolish the shedding of bacteria in the livestock reservoir. For instance, a vaccine, called Ome, developed using the type III secreted proteins decreased the shedding of STEC from 9% to 23%, and other vaccines which were developed using EspA, Tir, and intimin profoundly lowered EHEC O157 shedding from experimentally infected cattle [158]. However, non-O157 serovar-specific vaccines are lacking.

Foodborne contamination caused by the above-mentioned serogroups can be reduced by several physical procedures (e.g., pasteurization, irradiation) and chemical agents (e.g., chlorine dioxide; peroxyacetic acid; sodium hypochlorite; and organic acids, including lactic, acetic, and citric). Despite their effectiveness, these methods are not appropriate for all foods and have inherent disadvantages (change in sensory characteristics of food, toxicity, etc.). In light of this, the development of effective and safe alternative methods has currently gained increased attention [159].

In recent years, research has become increasingly focused on developing different efficient intervention strategies including bacteriophages, colicins, prebiotics, synbiotics, and others to reduce the burden of the disease. In addition, researchers have been investigating the mechanism of how the microbial population and host physiology influence STEC populations in cattle’s guts. The results of those research have led to several novel interventions as well as potential dietary additions or changes that can reduce STEC in animals, many of which have already entered the market or are in the process of doing so [1,160,161].

## 9. Concluding Remarks

The incidence of non-O157 STEC infections has been increased over the past decade and caused mild to severe diseases in humans. Due to the phenotypic and genetic variability of non-O157 *E. coli* serogroups, the development of reliable and accurate methods for in vivo and in vitro investigation of these pathogens has been challenging. Hence, intensive studies on their evolution, genetics, pathogenicity, and physiology are needed to develop efficient and successful control strategies. In addition, more emphasis has to be given to tackling the transmission of infectious agents from reservoir animals to humans. As part of foodborne infection, innovative intervention strategies should be designed to prevent the penetration of the infectious agent into the food chain. Public health professionals, epidemiologists, and other responsible bodies have to be encouraged to investigate the status of the disease and its antimicrobial resistance profile for better control of the disease.

Most of the diagnostic methods reviewed in this paper are labor intensive, time consuming, and require expensive equipment. Therefore, there is a necessity for sensitive, specific, accurate, user-friendly, deliverable, and rapid detection methods for reliable and efficient outcomes. Emerging technologies including biosensors, paper-based chips, CRISPR-Cas-based diagnosis, mobile PCR, isothermal amplification methods, surface-enhanced Raman spectroscopy, and smartphone-based digital technologies are novel approaches for the detection of O157:H7 serovars that can be also used for detection of non-O157 STEC serovars.

## Figures and Tables

**Table 1:** Major outbreaks caused by non-O157:H7 infections.

Year	Cause	Country	Sources of Infection	Outcomes	References
2019	O103 STEC	Multistate USA outbreak	Ground Beef	209 cases (29 hospitalizations and 2 HUS)	[123]
2015	STEC O26	USA	Mexican grill restaurant	55 cases (21 hospitalizations)	[124]
2015	STEC O26	USA	Mexican grill restaurant	Five illnesses	[124]
2011	EHEC O104:H4	Germany	Fenugreek seeds sprouts imported from Egypt	More than 4000 people (49 dead)	[125]
2010	O145	USA	Romaine Lettuce	26 confirmed and 7 probable cases	[126]
2012	O111:NM	Oklahoma USA	Cross-contamination between food preparation utensils or food handlers and restaurant food	341 cases (70 hospitalizations, and 1 death)	[127]
2014	O104:H4	Germany	Food handler contamination	23 cases in a family party	[128]
2014	O26	USA	Person to person spread (*n* = 15) or Food (*n* = 17)	38 single-etiology outbreaks (66% of the total cases)	[91]
2007	O26	Denmark	Beef sausage	20 cases	[129]
2007	O145, O26	Belgium	Ice cream	12 cases (5 HUS)	[116]
1995	O111:NM	Australia	Sausage	158 cases (23 HUS)	[130]
1999	O121	USA (CT)	Lake water	11 cases	[109]
1990	O111	USA (Ohio)	Undetermined	5 cases	[131]
1999	O111	USA (Texas)	Salad bar	56 cases	[89]

NM—nonmotile

## Data Availability

Not applicable.
